# Structural basis for defective membrane targeting of mutant enzyme in human VLCAD deficiency

**DOI:** 10.1038/s41467-022-31466-2

**Published:** 2022-06-27

**Authors:** Michelle S. Prew, Christina M. Camara, Thomas Botzanowski, Jamie A. Moroco, Noah B. Bloch, Hannah R. Levy, Hyuk-Soo Seo, Sirano Dhe-Paganon, Gregory H. Bird, Henry D. Herce, Micah A. Gygi, Silvia Escudero, Thomas E. Wales, John R. Engen, Loren D. Walensky

**Affiliations:** 1 Department of Pediatric Oncology, Dana-Farber Cancer Institute, Boston, MA USA; 2 Linde Program in Cancer Chemical Biology, Dana-Farber Cancer Institute, Boston, MA USA; 3 Department of Chemistry and Chemical Biology, Northeastern University, Boston, MA USA; 4 Department of Cancer Biology, Dana-Farber Cancer Institute, Boston, MA USA

**Keywords:** Protein folding, Molecular medicine, Proteomics, X-ray crystallography

## Abstract

Very long-chain acyl-CoA dehydrogenase (VLCAD) is an inner mitochondrial membrane enzyme that catalyzes the first and rate-limiting step of long-chain fatty acid oxidation. Point mutations in human VLCAD can produce an inborn error of metabolism called VLCAD deficiency that can lead to severe pathophysiologic consequences, including cardiomyopathy, hypoglycemia, and rhabdomyolysis. Discrete mutations in a structurally-uncharacterized C-terminal domain region of VLCAD cause enzymatic deficiency by an incompletely defined mechanism. Here, we conducted a structure-function study, incorporating X-ray crystallography, hydrogen-deuterium exchange mass spectrometry, computational modeling, and biochemical analyses, to characterize a specific membrane interaction defect of full-length, human VLCAD bearing the clinically-observed mutations, A450P or L462P. By disrupting a predicted α-helical hairpin, these mutations either partially or completely impair direct interaction with the membrane itself. Thus, our data support a structural basis for VLCAD deficiency in patients with discrete mutations in an α-helical membrane-binding motif, resulting in pathologic enzyme mislocalization.

## Abstract (web summary)

Prew et al. uncovered a structural basis for human VLCAD deficiency that arises from point mutations within the enzyme’s membrane-binding region, which was shown to fold as a putative α-helical hairpin. Helix-breaking mutations selectively disrupt membrane interaction and thus homeostatic function.

## Introduction

Fatty acid β-oxidation (FAO) is an essential metabolic pathway that involves sequential breakdown of fatty acids in the mitochondria to ultimately yield cellular energy in the form of ATP^1^. Depending on chain length, fatty acyl-CoAs can undergo the first step of β-oxidation by one of four acyl-CoA dehydrogenases (ACADs), namely short- (SCAD), medium- (MCAD), long- (LCAD), and very long- (VLCAD) chain enzymes. Whereas SCAD, MCAD, and LCAD operate as tetramers in the mitochondrial matrix and share considerable sequence homology^2^, VLCAD is an outlier that localizes to the inner mitochondrial membrane as a dimer and contains a unique additional stretch of 180 amino acids at its C-terminus^3–5^. Whereas short- and medium-chain fatty acids enter the mitochondria by diffusion, long-chain acyl-CoAs require conversion to acylcarnitine analogs by carnitine palmitoyltransferase 1 (CPT1), import by carnitine-acylcarnitine translocase (CACT), and ultimately reversion to the acyl-CoA forms by carnitine palmitoyltransferase 2 (CPT2)^6^. All three enzymes are membrane-associated^7^, likely explaining why the initial rate-limiting step of oxidizing fatty acids containing 14–20 carbon chains in length occurs in assembly line fashion by the inner mitochondrial membrane-associated enzyme, VLCAD.

The crystal structure of human VLCAD in complex with myristoyl-CoA^8^ revealed that Glu-422 functions as the key catalytic residue, analogous to Glu-376 in MCAD^9,10^, and that the selectivity for long-chain fatty acyl-CoAs is afforded by a wider opening of the substrate-binding pocket, which extends 12 Å beyond where the analogous pocket ends in MCAD. The unique C-terminal region is comprised of an α-helical bundle that lies perpendicular to the enzyme and further includes a non-visualized region spanning amino acids 446–478 believed to mediate membrane interaction. The mechanisms by which VLCAD engages the membrane and captures substrate for delivery to the binding pocket, either by the mobility of the juxta-membrane region and/or participation in an enzymatic complex, remain unresolved.

Defective or partially deleted VLCAD protein underlies VLCAD deficiency, an inborn error of metabolism that has diverse and potentially fatal manifestations due to crises in energy production during fasting or periods of increased metabolic demand^11–13^. VLCAD deficiency is the most common long-chain FAO disorder, with an incidence of 1 in 30,000–100,000^14^. The severe, early-onset form, which arises due to near absence of the enzyme, typically manifests within the first several months of life and is characterized by cardiac disease (cardiomyopathy, pericardial effusions, arrhythmias), hypotonia, hepatomegaly, episodic hypoglycemia, and a high mortality rate (75%). The medium-onset variant is milder, occurs in childhood, and manifests with hypoketotic hypoglycemia. The late-onset and mildest form can trigger rhabdomyolysis upon fasting or exercising. Early diagnosis and intervention are critical to avoid morbidity and mortality; sudden death has been reported in up to 52% of patients, typically due to cardiac conduction abnormalities. Newborn screening has been effective for early diagnosis^15^. Treatment principles focus on preventing and controlling acute episodes by avoiding periods of fasting and maintaining a low-fat, high-carbohydrate diet, including supplementation with medium-chain triglyceride supplements^16^. For acute, life-threatening crises, prompt intravenous glucose can be lifesaving. Of the series of VLCAD protein mutants implicated in enzymatic deficiency^17,18^, two human mutations that produce milder disease, A450P^19^ and L462P^17^, localize to the structurally undefined region of the C-terminus^20^. Thus, a structure-function dissection of this region has the potential to inform a mechanism of enzyme regulation through membrane interaction.

## Results

### A structurally-dynamic subdomain of the VLCAD C-terminus is required for direct membrane interaction

**Fig. 1:**
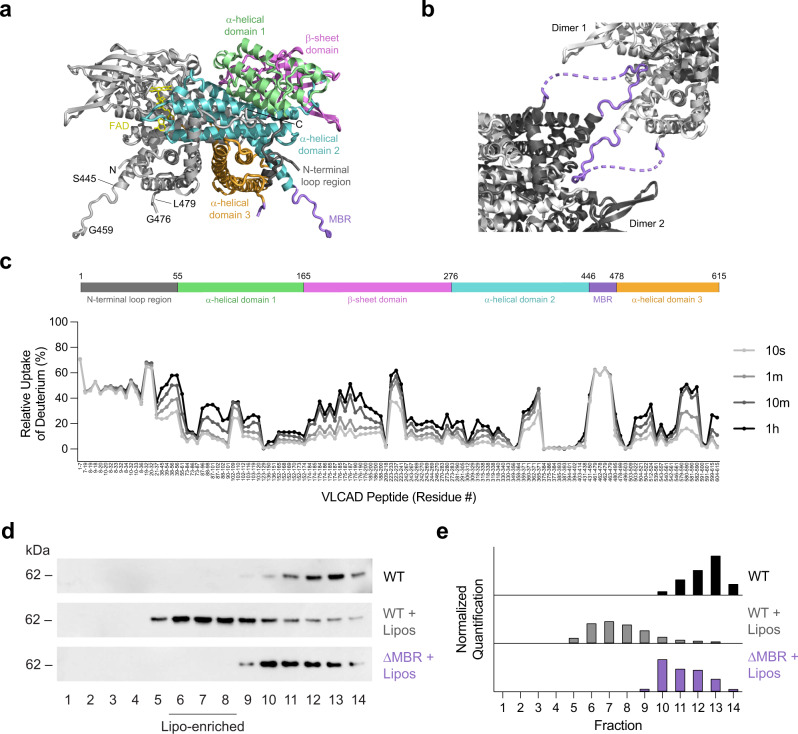
Structural and functional characterization of human VLCAD by X-ray crystallography, HDX MS analysis, and liposomal translocation assay. **a** Dimeric structure of human VLCAD ΔEx3 (PDB ID 7S7G) demonstrating its subcomponents and expanded MBR residues (purple) that extend outward from the protein core. See Supplementary Table 1 for data collection and results of refinement. **b** Crystal contacts between neighboring VLCAD dimers involve reciprocal interactions between the MBR of one dimer and the surface groove of another. **c** Relative deuterium uptake (%) of full-length VLCAD in solution after 10 s, 1 m, 10 m, and 1 h of deuteration. Residues are numbered using the mature (cleaved leader sequence) form of human VLCAD. HDX MS experiments were performed twice using independent preparations of VLCAD protein. See Supplementary Data 1 for the HDX MS data used to create this figure. **d** Translocation of VLCAD, but not its ΔMBR deletion mutant, to liposomes bearing the lipid composition of the inner mitochondrial membrane, as monitored by VLCAD western analysis of SEC fractions. The experiment was repeated twice with independent preparations of VLCAD protein with similar results. **e** Quantitation of VLCAD observed in liposomal translocation assay fractions (**d**) by densitometry using ImageJ software.

Two crystal structures of human VLCAD lacking exon 3 (VLCAD ΔEx3) have been solved to date, each missing electron density for a stretch of amino acids corresponding to the proposed membrane binding region (MBR) of VLCAD (PDB ID 3B96^8^, 446–478; PDB ID 2UXW, 457–476). In an effort to obtain further structural data, we generated recombinant human VLCAD ΔEx3 and solved a crystal structure with additional electron density in this region (PDB ID 7S7G) (Fig. 1a). Specifically, on the N-terminal side of the density gap, the structure demonstrated residues through amino acid 459 facing away from the protein (Fig. 1a and Supplementary Fig. 1a), consistent with a module capable of projecting outward to engage a membrane surface. No secondary structure was evident in this region and residues 460–475 remain unresolved, and thus highly flexible, in this structure generated in the solution phase. Of note, the crystal contacts demonstrated a reciprocal interaction between the outwardly projecting MBR of one VLCAD dimer and the surface groove of its neighboring dimer, consistent with a stabilizing interaction for this otherwise exposed and disordered region (Fig. 1b and Supplementary Fig. 1b). Although these data provided additional evidence of a conformationally flexible MBR in the absence of membrane, the limitations of crystallography, in this case, motivated our pursuit of alternative, multidisciplinary approaches to achieve additional structure-function insight.

To investigate the conformational dynamics of VLCAD, we generated the full-length protein and analyzed it by hydrogen-deuterium exchange mass spectrometry (HDX MS). HDX MS probes protein structure by measuring deuterium exchange of backbone amide hydrogens^21,22^. When diluted into deuterium buffer, backbone hydrogens of flexible and/or exposed protein regions rapidly exchange with deuterium. In contrast, buried domains and/or those regions that contain hydrogen bonding of backbone amide hydrogens (such as in α-helices) demonstrate slowed or suppressed deuterium exchange. The HDX MS profile of VLCAD in solution, evaluated after 10 s, 1 m, 10 m, and 1 h of deuteration (Fig. 1c), was consistent with the structural organization seen by X-ray crystallography, including general agreement between deuteration and crystallographic B factors (Supplementary Data 1)^8^. For example, the flexibility of the N-terminal loop region (aa 1–55) was evidenced by the high degree of deuterium uptake even at the earliest time points. In contrast, the various mainly structured regions, including the α-helical domain 1 (aa 56–165), β-sheet domain (aa 166–276), α-helical domain 2 (aa 277–445), and α-helical domain 3 (aa 479–615), exhibited an overall low level of deuterium uptake that only modestly increased in a time-dependent fashion. Peptides within these structured areas that did demonstrate higher deuterium uptake correspond primarily to loops. Notably, that portion of the C-terminus containing the MBR and not fully resolved by X-ray crystallography (e.g., aa 446–478) displayed a high degree of deuterium uptake from early to late labeling times, consistent with marked flexibility of this region in solution.

To evaluate whether the amino acids of this structurally-dynamic region of VLCAD are explicitly required for direct membrane interaction, we generated and tested full-length VLCAD and a deletion mutant lacking 23 residues of this region (VLCAD Δ450–472 or ΔMBR) in a liposomal translocation assay. Using liposomes reflecting the lipid composition of the inner mitochondrial membrane, namely 80% phosphatidylcholine (PC) and 20% cardiolipin (CL)^23–25^, we demonstrated that VLCAD, but not its ΔMBR mutant, was capable of translocating from solution to the liposomes, as monitored by size exclusion chromatography (SEC) (Fig. 1d) and quantitated by densitometry of the western blot of VLCAD-containing fractions (Fig. 1e). Membrane translocation was verified by documenting co-elution of full-length VLCAD protein with liposomes, which were tracked by phosphatidylcholine quantitation of each fraction. Whereas the SEC profiles of full-length VLCAD protein and liposomes alone were distinct and essentially non-overlapping (Supplementary Fig. 2a, b), upon combination, the VLCAD elution profile mirrored that of the liposomes (Supplementary Fig. 2c, d). To probe the membrane selectivity of VLCAD for cardiolipin-enriched liposomes reflective of the inner mitochondrial membrane (80% PC, 20% CL)^24^, we repeated the translocation experiment with liposomes that instead mimic the membrane composition of the outer mitochondrial membrane (48% PC, 28% phosphatidylethanolamine, 10% phosphatidylinositol, 10% phosphatidylserine, and 4% CL)^26,27^ and observed little to no translocation (Supplementary Fig. 2e, f). Taken together, the X-ray crystallography, HDX MS, and liposomal translocation assays indicate that, in solution, the structurally-unresolved portion of the C-terminal region is solvent-exposed, conformationally dynamic, and essential to VLCAD interaction with cardiolipin-enriched membrane.

### Proline mutations observed in human VLCAD deficiency selectively impair membrane interaction

**Fig. 2:**
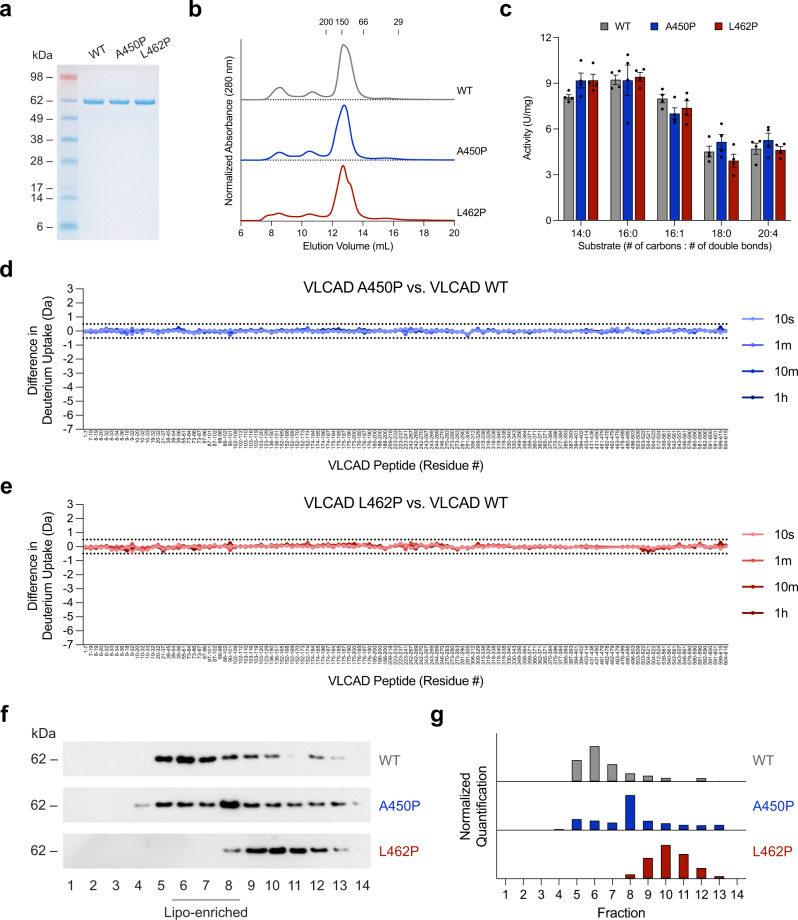
Comparative SEC, enzymatic activities, HDX MS profiles, and membrane interaction analyses of wild-type VLCAD protein and two proline-mutant variants observed in human VLCAD deficiency. **a** SDS-PAGE and Coomassie stain of expressed and purified full-length VLCAD protein and its A450P and L462P mutants. The experiment was repeated twice using independent preparations of VLCAD proteins with similar results. **b** SEC elution profiles of wild-type, A450P, and L462P VLCAD proteins. **c** Comparative enzymatic activities of wild-type, A450P, and L462P VLCAD proteins, as assessed using a series of long-chain substrates and ferrocenium hexafluorophosphate as the electron acceptor. Data are mean ± s.e.m. for experiments performed in technical quadruplicate and repeated twice with independent preparations of assay reagents with similar results. **d**, **e** Deuterium difference plots showing the relative deuterium incorporation in solution of VLCAD A450P (**d**) and L462P (**e**) minus the relative deuterium incorporation of wild-type VLCAD, as measured after 10 s, 1 m, 10 m, and 1 h of deuteration. Regions of deprotection and protection above 0.5 Da (dotted line) are considered meaningful. HDX MS experiments were performed twice with independent preparations of VLCAD proteins. See Supplementary Data 1 for the HDX MS data used to create this figure. **f** Comparative liposomal translocation of wild-type, A450P, and L462P VLCAD proteins, as monitored by VLCAD western analysis of SEC fractions. The experiment was repeated twice with independent preparations of VLCAD proteins with similar results. **g** Quantitation of VLCAD observed in liposomal translocation assay fractions (**f**) by densitometry using ImageJ software.

Two proline mutations linked to human VLCAD deficiency, A450P and L462P, occur within the MBR. To examine the impact of these mutations on the structure and function of VLCAD, we generated and characterized each proline mutant, in full-length form, in a series of assays. First, we found that all three constructs could be expressed and purified in similar yield and stability and eluted as a monodispersed peak corresponding to the native dimeric conformer (~150 kDa) (Fig. 2a, b). Second, we evaluated comparative enzymatic activities using a series of long-chain substrates and ferrocenium hexafluorophosphate as the electron acceptor^28^. Upon exposure to substrate, the decrease in ferrocenium absorbance was recorded over time, and activity calculated in units of U/mg. Notably, proline mutagenesis had no effect on enzymatic activity in solution (Fig. 2c and Supplementary Fig. 3). In a prior study, similar activity between wild-type and L462P VLCAD proteins was observed using crude protein extracts from *E. coli* but upon protein purification, the L462P variant was less active, potentially due to relatively decreased enzyme stability^20^. Other differences between the current analysis and prior work include our use of full-length rather than ΔEx3 VLCAD proteins, and a different electron acceptor (ferrocenium hexafluorophosphate vs. native and purified electron transfer flavoprotein)^20^. Third, we compared the HDX MS profiles of the two proline mutants to that of wild-type VLCAD and observed no differences in deuterium exchange in solution (Fig. 2d, e). Finally, to investigate the structure-function consequences of proline mutagenesis in the membrane context, we performed a comparative liposomal translocation assay. Whereas wild-type VLCAD demonstrated near complete membrane translocation, the A450P variant exhibited only partial liposomal association, and the L462P mutant showed essentially no translocation activity, as assessed by SEC (Fig. 2f) and quantitated by densitometry of the western blot of VLCAD-containing fractions (Fig. 2g). These data indicate that proline mutagenesis has little to no effect on enzymatic stability, dimeric assembly, enzymatic activity, or overall conformation of VLCAD in solution, but selectively impairs membrane interaction.

**Fig. 3:**
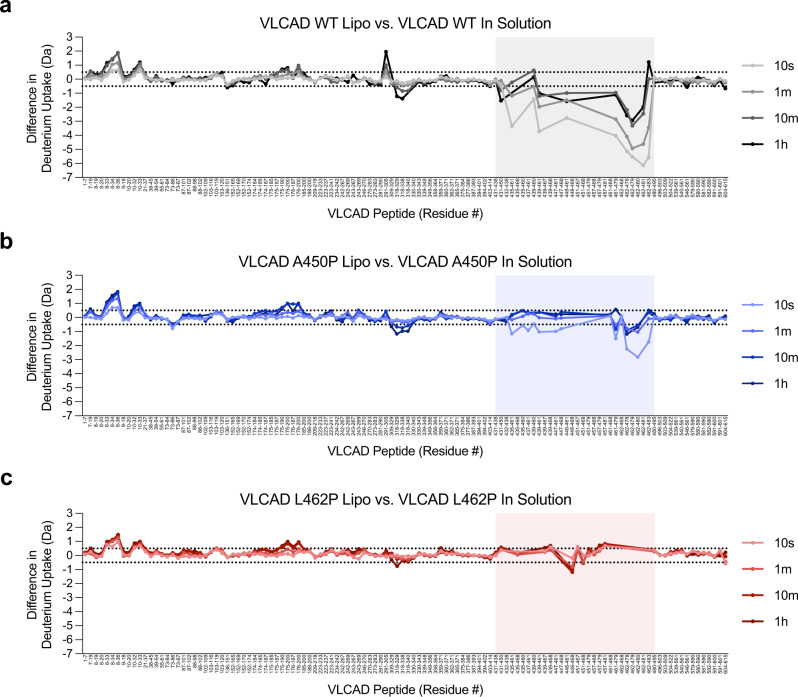
HDX MS analysis of wild-type and proline-mutant VLCAD proteins in the presence and absence of liposomal membranes. **a**-**c** Deuterium difference plots showing the relative deuterium incorporation of wild-type (**a**), A450P (**b**), and L462P (**c**) VLCAD proteins in the presence of liposomes minus the relative deuterium incorporation in solution, as measured after 10 s, 1 m, 10 m, and 1 h of deuteration. Regions of deprotection and protection above 0.5 Da (dotted line) are considered meaningful. Peptides of the VLCAD C-terminal region that show protection in the presence of liposomal membrane, an effect notably impacted by proline mutagenesis, are shaded. HDX MS experiments were performed twice with independent preparations of VLCAD proteins. See Supplementary Data 1 for the HDX MS data used to create this figure.

To monitor the effect of lipid membrane on the structural dynamics of wild-type and mutant VLCAD, we performed HDX MS analysis in the presence and absence of liposomes that mimic the lipid composition of the inner mitochondrial membrane. In the presence of liposomes, we observed striking protection from exchange of amino acids 450–479 in wild-type VLCAD, especially evident at the earlier deuteration time points of 10 s and 1 m (Fig. 3a). The protection stemmed from the formation of a new and less-deuterated population (an EX1 kinetic signature in HDX MS) in the presence of liposomes, consistent with induction of helical structure in the 450–479 region upon membrane interaction (Supplementary Fig. 4). In accordance with the liposomal translocation data, the A450P and L462P mutants showed decreased protection in the 450–479 region relative to wild-type VLCAD in the presence of liposomes (Fig. 3b, c). Correspondingly, little to no EX1 kinetic signatures were observed for the proline mutants (Supplementary Fig. 4). The data collectively indicate that the structure of this discrete region of the VLCAD C-terminus is dramatically influenced by the membrane environment, an effect that is selectively impaired by proline mutagenesis. From a mechanistic standpoint, this striking level of backbone amide shielding in the presence of liposomes is consistent with membrane insertion and/or induced folding of the previously unstructured region upon membrane interaction.

### A mechanism of VLCAD deficiency based on proline-induced disruption of a membrane-binding α-helical hairpin

**Fig. 4:**
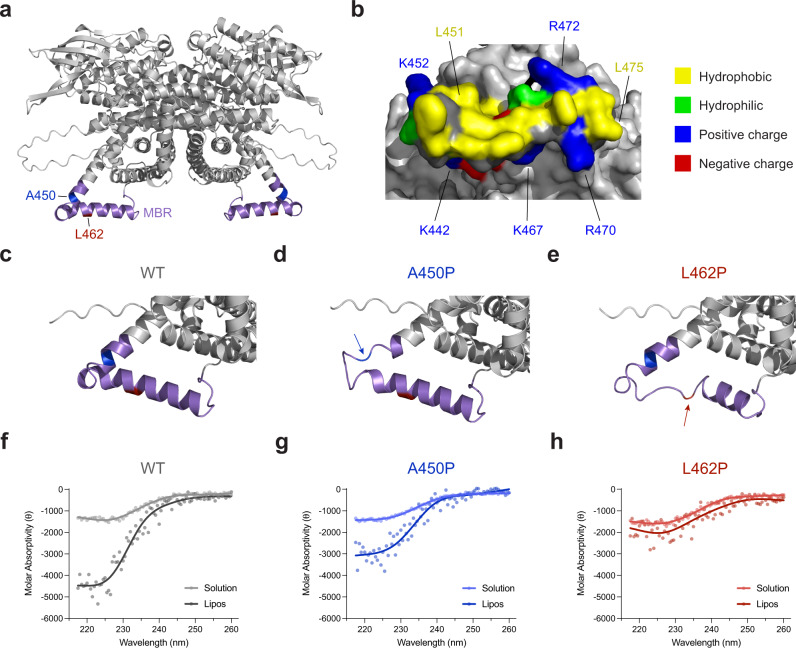
Computational and circular dichroism analyses support a model for proline-mediated disruption of a membrane-interacting hairpin located within VLCAD’s C-terminal domain. **a** AlphaFold model structure of dimeric VLCAD demonstrating residues 440–473 as a helix-turn-helix hairpin. Residues A450 and L462, two sites of proline mutagenesis in human VLCAD deficiency, are colored in blue and red, respectively. **b** A surface view of the predicted α-helical hairpin demonstrates a hydrophobic interface surrounded by a perimeter of positively-charged residues, including lysines 440, 442, 452, 467 and arginines 470, 471, 472. **c**-**e** Model structures (AlphaFold) for wild-type (**c**), A450P (**d**), and L462P (**e**) VLCAD proteins demonstrate how proline mutagenesis can disrupt the structure of the α-helical hairpin. **f**-**h** Circular dichroism (CD) of wild-type (**f**), A450P (**g**), and L462P (**h**) peptides corresponding to residues 440–473 of the predicted α-helical hairpin in solution and in the presence of liposomes (relative α-helicity in the presence of liposomes of 2.5:1.7:1 for wild-type:A450P:L462P). CD experiments were performed in duplicate using independent preparations of peptides with similar results.

The release of AlphaFold (DeepMind)^29^, an artificial intelligence-based predictor of protein folding, allowed us to map the very region of VLCAD that is protected from deuterium exchange upon membrane exposure onto a model structure. Interestingly, the structurally unresolved portion of the C-terminal region (e.g., aa 446–478) is predicted to fold as a helix-turn-helix hairpin based on sequence homology to structured proteins in the database (Fig. 4a). We found that deletion of one or the other α-helix of the putative hairpin markedly reduced liposomal membrane translocation of VLCAD (Supplementary Fig. 5a–c), highlighting the potential importance of this secondary structure. A surface view of the region demonstrates a prominent hydrophobic interface bordered by a series of positively-charged residues, suggesting a mechanism of membrane engagement that involves hydrophobic anchoring reinforced by electrostatic interaction with negatively-charged lipid head groups (e.g., cardiolipin) (Fig. 4b and Supplementary Fig. 5d). Intriguingly, HDX MS analysis of VLCAD in the presence of liposomes but at a higher osmolyte concentration^30^ (500 mM KCl vs. 200 mM KCl) revealed a decreased magnitude of protection from deuterium exchange in the 450–479 region (Supplementary Fig. 6), consistent with decreased membrane residence time due to osmolyte interference with ionic interactions and/or α-helical induction. Based on the folding prediction, each of the two proline mutations localizes to one or the other α-helix of the putative hairpin (Fig. 4a). A proline mutation in this region would be expected to disrupt α-helical folding^31^ and preclude an alignment of the hydrophobic and positively-charged residues necessary for optimal membrane binding. Although AlphaFold has not been validated for predicting the structural effects of point mutations, modeling the A450P and L462P point mutations in AlphaFold demonstrates disruption of α-helical structure in the vicinity of the proline mutation, consistent with the role of proline as a helix breaker^31^ (Fig. 4c–e and Supplementary Fig. 7).

To test the model of MBR structure, we synthesized wild-type as well as A450P and L462P mutant peptides corresponding to the proposed α-helical hairpin of VLCAD (aa 440–473) (Supplementary Fig. 8a) and performed HDX MS analysis to measure deuterium exchange in solution upon increasing percentages of trifluoroethanol (TFE), a solvent that can induce helical folding. Whereas the wild-type and A450P peptides demonstrated reduced deuterium exchange with increasing TFE, consistent with at least partial helical folding, the L462P mutant remained more unstructured, as evidenced by comparatively more deuterium exchange at higher TFE percentages (Supplementary Fig. 8b). We then subjected the three peptides to circular dichroism analysis in the presence and absence of liposomes to specifically compare induction of α-helical structure upon membrane exposure. We found that the random coil structure of the wild-type peptide in solution became more α-helical in the presence of liposomes (Fig. 4f), with the A450P mutation having an intermediate effect (Fig. 4g) and the L462P mutation resulting in no conformational change (Fig. 4h). Thus, the circular dichroism data validate the AlphaFold models (Fig. 4c–e) and are remarkably consistent with the stepwise decrement observed in membrane translocation upon A450P and L462P mutagenesis of wild-type VLCAD protein (Fig. 2f, g).

### Toward a structural model of membrane-anchored VLCAD

**Fig. 5:**
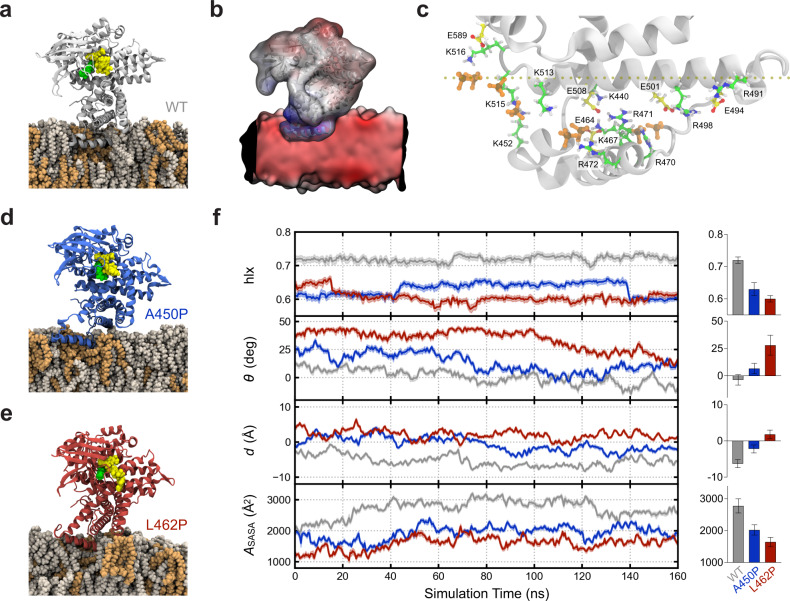
Molecular dynamics simulations of VLCAD and its proline mutants in the presence of membrane. **a** Simulated structure of a wild-type VLCAD monomer in contact with a cardiolipin-containing membrane bilayer (4:1 ratio of phosphatidylcholine [POPC]: tetraoleoyl cardiolipin [TOCL]), as demonstrated by molecular dynamics simulation after 125 ns of equilibration. Residues 446–474 of the α-helical hairpin insert deeply and stably into the lipid membrane. POPC, tan; TOCL, orange; FAD, yellow; substrate, green. **b** Independently computed electrostatic maps for wild-type VLCAD and lipid membrane, illustrating a complementary potential *V* between adjacent regions (blue: *V* > 0; red: *V* < 0; white, *V* = 0). **c** Representative electrostatic interactions between cationic (K, R) residues of VLCAD and cardiolipin headgroups of the lipid membrane or anionic (D, E) amino acids of the protein. To maintain clarity, only the charged phosphates (PO_4_^2−^) of TOCL (orange) are shown. **d**, **e** Proline mutagenesis disrupts the local structure, resulting in more superficial membrane interaction compared to wild-type VLCAD, as demonstrated by molecular dynamics simulation of the A450P and L462P variants after 125 ns of equilibration. **f** Comparative geometric quantification of the membrane interactions of wild-type and proline-mutant VLCAD proteins, including, from top to bottom, (1) helical content hlx of the C-terminal region comprised of amino acids 436–488, (2) instantaneous tilt angle *θ* between a membrane-embedded α-helical segment (residues 460–474) and the membrane surface, (3) height *d* of the helix center-of-mass with respect to the membrane surface, as defined by a plane through phosphate atoms in the proximal membrane leaflet, whereby values with *d* < 0 lie below the lipid headgroups, and (4) solvent-accessible surface area *A*_SASA_ buried at the VLCAD-lipid interface. Trajectories were smoothed by averaging over 0.5 ns bins, and bands reflect the standard deviation within these bins. Calculations were facilitated by the collective variables module of VMD^71^ where applicable^72^. Adjacent bar graphs depict mean values ± s.d. of the geometric parameters, calculated over the last 80 ns equilibration trajectory (*n* = 320).

To build on our collective findings and develop a structural model for VLCAD-membrane interaction, we performed molecular dynamics simulations of a VLCAD monomer in contact with a model membrane containing cardiolipin (4:1, PC:CL). We observe that residues 446–474 of the α-helical hairpin at the VLCAD C-terminus rapidly become firmly embedded within the membrane. The orientation of the α-helical hairpin within the membrane is supported by an extensive network of complementary hydrophobic and electrostatic interactions (Fig. 5a, b). Notably, establishing an initial position of dimeric VLCAD in contact with the membrane (Supplementary Fig. 9a) revealed an orientation consistent with that of monomeric VLCAD, highlighting the utility of simulating the monomer as a proxy for dimer dynamics, particularly with respect to generating hypotheses regarding local MBR structure and interactions (Supplementary Fig. 9b, c). Indeed, these simulations uncovered a series of stabilizing salt bridges between cationic sidechains of VLCAD and (1) anionic lipid headgroups (e.g., R470, R472, K513, K515, K516, and R592), and (2) anionic residues within the protein itself (e.g., K467, R471, R491, R498, and R592) (Fig. 5c and Supplementary Fig. 10). In contrast, simulations performed with VLCAD bearing the A450P or L462P mutations demonstrate alterations in this interaction network and notably decreased membrane engagement (Fig. 5d, e and Supplementary Fig. 10).

To rigorously compare the simulated protein-membrane interactions of VLCAD and its proline-mutant variants, we performed quantitative analyses using a series of geometric parameters. Using a collective variable hlx^32^, which captures the relative helical content of a polymer and serves as an order parameter for the helix-coil transition^33,34^, we found that the α-helical character of C-terminal residues 436–488 in the wild-type protein remained high throughout the simulation, whereas the α-helical content of the corresponding region in the A450P and L462P variants was markedly decreased during the initial equilibration (Fig. 5f). This loss of secondary structure persisted for the L462P mutant, while the A450P variant underwent switching between conformational states of varying α-helical content, all of which remained less helical than that observed for wild-type VLCAD. We next quantified the membrane orientation of the VLCAD MBR using the tilt angle *θ* between the stable 460–474 α-helix and the bilayer plane. For wild-type VLCAD, this α-helical segment became oriented parallel to the bilayer surface and remained in this position with high temporal stability. In contrast, the proline mutants required a prolonged timescale to approach an analogous orientation (Fig. 5f). We examined this phenomenon further by measuring the height *d* of the 460–474 α-helix center-of-mass with respect to the leaflet surface, as defined by a plane through the phosphate headgroups. In wild-type VLCAD, the α-helix was deeply embedded in the membrane as a rigid body (Fig. 5a, b, f), whereas proline-induced fraying of the secondary structure precluded this insertion behavior for the mutant variants, as reflected by their higher *d* values. The fixed orientation of the 460–474 α-helix and its depth of membrane insertion collectively pulled the body of wild-type VLCAD closer to the membrane, as indicated by the large protein solvent-accessible surface area *A*_SASA_ that becomes buried in the protein-membrane complex (Fig. 5f). Conversely, the geometry conferred by proline mutagenesis resulted in a larger, solvent-accessible void space that reduced buried contacts. Since the hydrophobic contributions to membrane binding are expected to scale linearly with buried surface area, the observed reduction in *A*_SASA_ values for the A450P and L462P variants is consistent with disruption of VLCAD-membrane interaction by proline mutagenesis. Taken together, the simulation data and our experimental results point to a mechanism of human VLCAD deficiency that derives from the conversion of hydrophobic residues located within an α-helical hairpin into helix-breaking prolines, which disrupt the conformation and complementarity of a C-terminal subregion critical to inner mitochondrial membrane interaction.

## Discussion

VLCAD is unique among the ACAD family in residing as a dimer at the inner mitochondrial membrane rather than localizing to the mitochondrial matrix^5^. Whereas short- and medium-chain fatty acids can freely diffuse into the mitochondrial matrix and undergo activation and oxidation by the corresponding matrix-localized acyl-CoA synthetase and ACAD, respectively, long-chain acyl-CoAs require specialized processing^6^. Specifically, long-chain acyl-CoAs are transiently converted to carnitine analogs for translocation across the inner mitochondrial membrane and then undergo oxidation by membrane-localized proteins including VLCAD, which serves as the most afferent and rate-limiting enzyme in the long-chain FAO pathway. Indeed, this juxta-membrane shuttling of long-chain fatty-acid substrates and their intermediates is believed to effectively redress their inherent insolubility^35^. The physiologic relevance of VLCAD’s discrete intracellular localization is underscored by the occurrence of clinically-relevant VLCAD deficiency when mutations preclude tethering to the inner mitochondrial membrane^20^.

Here, we find that a structurally-unresolved portion of the unique 180 amino acid C-terminal extension of VLCAD not found in other ACADs is required for direct membrane interaction. Although highly flexible in solution, the MBR region of VLCAD undergoes a dramatic conformational change and α-helical folding in the presence of cardiolipin-enriched membrane, as respectively demonstrated by HDX MS and circular dichroism analyses. Computational modeling further suggested that, in a membrane environment, discrete residues of the MBR can form an α-helical hairpin comprised of a hydrophobic surface surrounded by positively-charged residues—features that are especially compatible with insertion into negatively charged membranes like the inner mitochondrial membrane. Indeed, membrane-induced folding of amphipathic peptides comprised of hydrophobic and positively charged residues maximizes membrane interaction of bioactive peptides such as melittin and other antimicrobial peptides^32,36^. Mutagenesis of the VLCAD MBR, whether by partial deletion (e.g., Δ450–472) or replacement of specific hydrophobic residues with helix-breaking prolines, as observed for the human A450P and L462P mutations, impair if not completely abrogate α-helical folding and membrane interaction. Importantly, the deficiency in membrane association is selective, as the recombinant, full-length variants were found to have equivalent expression, stability, and enzymatic activity to the wild-type enzyme.

The spectrum of clinical manifestations associated with VLCAD deficiency, including life-threatening episodes of hypoketotic hypoglycemia and potentially fatal cardiac abnormalities, have long compelled rigorous investigations of the structure and function of VLCAD and its modes of regulation. We recently reported that the mitochondrial matrix isoform of MCL-1 (MCL-1^Matrix^), which resides at the inner mitochondrial membrane, directly interacts with VLCAD^37^. The results of photoaffinity labeling and HDX MS analyses were consistent with a helix-in-groove mode of interaction between the MCL-1 BH3 domain and a surface groove of VLCAD comprised of the α-helical domain 2, which lies proximal to the binding sites for the FAD cofactor and acyl-CoA substrate. Selective elevation of long-chain fatty acylcarnitines was observed upon deletion of the MCL-1^Matrix^ isoform in cultured fibroblasts and in *Mcl-1*-deleted murine livers^37^, consistent with deregulated long-chain fatty acid oxidative flux. SIRT3 and SIRT5 have also been shown to regulate mitochondrial FAO by respectively deacetylating and desuccinylating key lysine residues involved in (1) stabilizing the essential FAD cofactor in the VLCAD active site and (2) regulating VLCAD interaction with mitochondrial membrane cardiolipin^25^. Strikingly, acetylation or succinylation of K442, K452, and K467—residues that otherwise confer positive charge to the proposed juxtamembrane region of the VLCAD C-terminus—abrogates mitochondrial membrane binding. SIRT3 deacetylation (K467) or SIRT5 desuccinylation (K442, K452, and K467) restores cardiolipin interaction. These data are consistent with the contribution of circumferential positive charge to α-helical hydrophobic surfaces in effectively engaging the negatively-charged inner mitochondrial membrane. Thus, disrupting the architecture or biophysical features of the putative membrane-binding α-helical hairpin of VLCAD, whether by the helix-breaking proline mutagenesis observed in patients or regulatory lysine acylation, causes functional impairment of VLCAD due to a consequential intraorganellar mislocalization.

## Methods

### Recombinant VLCAD expression and purification

His-tagged human VLCAD lacking its cleavable leader sequence (pET19b vector, Millipore) was expressed in BL21(DE3) *E. coli* (Invitrogen) at 37 °C. Protein expression was induced at an O.D. of 0.8 with 0.5 mM IPTG for 4 h. Bacterial pellets were resuspended in lysis buffer (500 mM NaCl, 50 mM HEPES, 5% glycerol, pH 7.5, complete protease inhibitor tablet [Roche]) followed by microfluidization (M-110L, Microfluidics) and centrifugation at 48,384 × *g* for 45 min. Cleared lysate was initially subjected to Ni-NTA (Qiagen) affinity purification chromatography, eluting with 300 mM imidazole and dialyzing overnight at 4 °C into a 250 mM NaCl, 50 mM Tris, pH 8.0 buffer. SEC using a Superdex 200 10/300 GL column (GE Healthcare) was then performed at 4 °C using the same buffer. Protein identification and purity was confirmed by SDS-PAGE followed by Coomassie staining and VLCAD western blot analysis (Cat# PA5–29959, RRID: AB_2547433, Thermo Fisher Scientific). VLCAD ΔMBR (lacking aa 450–472), A450P, L462P, ΔH1 (lacking aa 446–454), and ΔH2 (lacking aa 455–478) mutants were generated by site-directed mutagenesis (Q5 Kit, New England Biolabs) using the following primers: GCAGGCCTGGGTAGCGGTCTGAG (F) and ACTGCCCAGGCCGCTCAGCTC (R) for ΔMBR, CCTGGGCAGTCCGCTGAAGAATCC (F) and CCGCTCAGCTCCTTACCC (R) for A450P, AGGTCTGCTGCCGGGCGAAGCAG (F) and GCGTTGCCAAACGGATTCTTCAG (R) for L462P, TTTGGCAACGCAGGTCTG (F) and GCTCAGCTCCTTACCCTTATC (R) for ΔH1, and CTGAGTTTAAGTGGCCTG (F) and CGGATTCTTCAGTGCACTG (R) for ΔH2. All protein variants were expressed, purified, and characterized as described above.

### Peptide synthesis

Peptides corresponding to amino acids 440–473 of human VLCAD and A450P and L462P variants were generated using solid-phase Fmoc chemistry on a Symphony X peptide synthesizer (Gyros Protein Technologies), with amino acids sequentially added to rink amide AM resin. Following N-terminal acetylation, deprotection, and cleavage from the resin, peptides were purified by reverse-phase high performance liquid chromatography and mass spectrometry (LC-MS) and quantified by amino acid analysis.

### X-ray crystallography

A variant of VLCAD lacking exon 3 (VLCAD ΔEx3) was previously developed to maximize bacterial expression and applied in X-ray crystallography studies^8,20^. VLCAD ΔEx3 was produced using the method described above for recombinant VLCAD proteins, followed by buffer exchange into 150 mM NaCl, 20 mM HEPES pH 7.5. Crystals were generated by the hanging drop method under 5 M NaCl, 1 M HEPES, 1 M DTT, pH 7.5 buffer conditions, and subsequently flash-frozen in liquid nitrogen. Diffraction data were collected at the Argonne National Laboratory synchrotron. The structure was solved to 1.34 Å (PDB ID 7S7G) by molecular replacement using PDB ID 3B96 and refined by Phenix and Coot software. Parameters for data collection and results of refinement are summarized in Supplementary Table 1.

### Hydrogen-deuterium exchange mass spectrometry

In addition to the summary descriptions below, comprehensive experimental details and parameters in the recommended tabular format^38^, proteolytic maps for all proteins, and the numeric values used to create the HDX MS figures are provided in Supplementary Data 1.

VLCAD proteins (1 μL at 40 μM) in the presence or absence of liposomes composed of 80% phosphatidylcholine and 20% cardiolipin (Cat# CAR-206, Encapsula Nanosciences) (1 μL of 10 mM HEPES, 200 mM KCl, 5 mM MgCl_2_, pH 7.0 or of 10 mM liposomes for 250-fold excess) were equilibrated at room temperature for 1 h. For the high osmolyte condition, experiments were performed with 1 μL of 20 μM VLCAD, 1 μL of 5 mM liposomes (250-fold excess), and buffer composed of 10 mM HEPES, 500 mM KCl, 5 mM MgCl_2_, pH 7.0. Deuterium labeling was initiated with an 18-fold dilution into labeling buffer at pD 7.0. After labeling for 10 s, 1 m, 10 m, or 1 h, the reaction was quenched with the addition of an equal volume of quenching buffer (pH 2.4). Samples were injected and digested online using a pepsin column (2.1 mm × 50 mm, pepsin immobilized on POROS-20AL beads). The peptides were trapped and desalted on a VanGuard Pre-Column trap (2.1 mm × 5 mm, ACQUITY UPLC BEH C18, 1.7 µM) for 3 min, eluted from the trap using a 5–35% acetonitrile gradient over 12 min at a flow rate of 100 μL/min, and then separated using a Waters nanoACQUITY LC equipped with an ACQUITY UPLC HSS T3, 1.8 µM, 1.0 mm × 50 mm column. The Waters Synapt G2Si mass spectrometer was operated in ion mobility mode. Peptides from an unlabeled sample were identified using ProteinLynx Global Server (PLGS, Waters) searches of a protein database containing the sequence of human VLCAD. Mass spectra were processed using DynamX 3.0 (Waters). Relative deuterium uptake for each peptide was calculated by subtracting the mass of the undeuterated control peptide from that of the labeled peptide. Deuterium levels were not corrected for back exchange and thus reported as relative^39^.

For analysis of exchange into the wild-type, A450P, and L462P VLCAD peptide constructs (Supplementary Fig. 8b), the synthetic peptides were dissolved in equilibration buffer (10 mM HEPES pH 7.2, 150 mM KCl) at 20 μM and kept on ice. The requisite amounts of TFE were added to achieve final mixtures of 0–75% TFE by volume and the pH re-verified and adjusted to 7.2 if needed. Deuterium labeling was initiated with an 18-fold dilution into D_2_O buffer (10 mM HEPES, pD 7.18, 150 mM KCl) that contained the same concentration of TFE as in the equilibration buffer solutions. Labeling proceeded for 10 s and was quenched with the addition of an equal volume of quench buffer (150 mM sodium phosphate pH 2.48). Samples were then injected onto an in-house packed POROS 20-R2 trap for peptide trapping and desalting for 3 min. A Waters nanoACQUITY LC was used to elute each peptide from the trap with a 15–70% gradient of acetonitrile over 6 min at a flow rate of 100 µL/min. Eluant was directed into a Waters Synapt G2si mass spectrometer operated in TOF-only mode for mass analysis. Data were analyzed as described^22,40^. All mass spectra were processed manually using MagTran. The relative amount of deuterium in the VLCAD constructs was determined by subtracting the centroid mass of the undeuterated form from the deuterated form. Deuterium levels were not corrected for back exchange and thus reported as relative^39^.

### Liposomal translocation assay

VLCAD proteins (0.5 μM) were mixed with liposomes (88 μM) composed of 80% phosphatidylcholine and 20% cardiolipin (Cat# CAR-206, Encapsula NanoSciences), reflecting the lipid composition of the inner mitochondrial membrane (IMM), in a total volume of 250 μL PBS, followed by incubation at room temperature for 1 h. The reaction mixtures were subjected to SEC using a Sepharose CL-2B column (GE Healthcare). Fourteen fractions (250 μL each) were collected and subjected to SDS-PAGE and VLCAD western blotting (Cat# PA5–29959, RRID: AB_2547433, Thermo Fisher Scientific). Densitometry analyses were performed using ImageJ v. 1.53 software. Verification of liposome-containing fractions was performed using the Phosphatidylcholine Assay Kit (Cat# MAK049, Sigma-Aldrich) according to the manufacturer’s instructions. Liposomes of alternative composition that mimic the lipid composition of the outer mitochondrial membrane (OMM) were generated by dissolving a 48:28:10:10:4 molar ratio of phosphatidylcholine, phosphatidylethanolamine, phosphatidylinositol, dioleoyl phosphatidylserine, and tetraoleoyl cardiolipin (Avanti Polar Lipids) in chloroform. Lipid films were produced by evaporating the chloroform using nitrogen gas and drying under high vacuum overnight, followed by storage in nitrogen at −80 °C. Lipid films were resuspended in a 10 mM HEPES, 200 mM KCl, 5 mM MgCl_2_, pH 7.0 buffer containing the fluorophore 8-aminoapthalene-1,3,6-trisulfonic acid (ANTS; 12.5 mM) and quencher p-xylene-bis-pyridinium bromide (DPX; 45 mM). Liposomes were generated by subjecting the resuspended lipid mixture to ten freeze/thaw cycles and extruding the liposomes using a 100 nm polycarbonate membrane eleven times. Liposomes were subsequently purified using a Sepharose CL-2B column (GE Healthcare). The translocation assay with OMM liposomes (40 μL) was performed as described above except that the 10 mM HEPES, 200 mM KCl, 5 mM MgCl_2_, pH 7.0 buffer was used instead of PBS for the incubation and fraction collection steps. Liposome-enriched fractions were identified by adding 10% Triton X-100 and measuring fluorescence from ANTS/DPX release.

### VLCAD enzymatic activity assay

Ferrocenium hexafluorophosphate (Sigma-Aldrich) was dissolved in 10 mM HCl to achieve a concentration of 1 mM. VLCAD protein was mixed with ferrocenium in 337.5 μL of assay buffer (100 mM potassium phosphate, pH 7.2, 0.1 mM EDTA) to achieve concentrations of 1.44 and 300 μM of protein and electron acceptor, respectively. Each reaction was initiated by adding 75 μL of 600 μM substrate (myristoyl-CoA [14:0], palmitoyl-CoA [16:0], palmitoleoyl-CoA [16:1], stearoyl-CoA [18:0], or arachidonoyl-CoA [20:4] [Sigma-Aldrich]) in assay buffer to 75 μL of the protein/ferrocenium mixture, resulting in dilution of each component by half, such that the final concentrations were 0.72 μM VLCAD, 150 μM ferrocenium, and 300 μM substrate. The decrease in ferrocenium absorbance at 300 nm was then measured as a function of time. Activity was calculated in units of U/mg based on the slope of the linear portion of the enzyme kinetic curves, as determined by Prism 9 software (GraphPad) using the molar absorptivity of ferrocenium (*ε* = 4.3 mM^−1^cm^−1^ at 300 nm).

### AlphaFold analysis

Predicted structures of VLCAD and its A450P and L462P mutant variants were determined by ColabFold: AlphaFold2^41^ using MMseqs2 (https://github.com/sokrypton/ColabFold) and standard parameters with Colab Pro+ set to a high-RAM GPU.

### Circular dichroism

Acetylated peptides were dissolved in PBS to achieve a concentration of 100 μM. Circular dichroism (CD) spectra were acquired in solution or in the presence of liposomes (80% phosphatidylcholine/20% cardiolipin; Cat# CAR-206, Encapsula NanoSciences) at a ratio of 1:50 on a spectropolarimeter (Aviv) using standard measurement parameters of temperature, 25 °C; step resolution, 0.5 nm; speed, 20 nm min^−1^; scans, 5. Signals at 217 nm and below were not recorded due to light scattering induced by the liposomes.

### Molecular dynamics simulations

Molecular dynamics simulations were constructed using an AlphaFold model for VLCAD lacking its cleavable leader sequence at the N terminus^29^. This prediction mimics existing crystal structures (RMSD = 0.194 Å vs. PDB ID 3B96 and RMSD = 0.208 Å vs. PDB ID 2UXW). Simulation physics was described using the CHARMM36 protein^42–45^ and lipid^46,47^ force fields. Bilayers containing a 4:1 ratio of phosphatidylcholine (POPC) to tetraoleoyl cardiolipin (TOCL^2−^) were constructed using the CHARMM GUI membrane builder^47–50^. We used dianionic parameters for TOCL, as indicated by prior biophysical measurements^51,52^. The VLCAD model was oriented by aligning its smallest principal moment of inertia normal to the lipid bilayer. Placement on the membrane was guided by electrostatic complementarity and experimental data^25^, which suggest that the 446–474 stretch might reside near lipid headgroups (lipid within 0.8 Å of protein atoms was removed in making this arrangement). Since the AlphaFold structure resembles post-catalytic VLCAD^8^, we stabilized this conformation by including an FAD cofactor and the partially-hydrolyzed trans(2)-tetradecanoyl-CoA product from crystal structures. These ligands were parameterized from CHARMM36 and CGenFF^53^. The simulation assembly was solvated normal to the membrane with TIP3P water^54–56^ (15 Å padding) and supplemented with 0.20 M K^+^/Cl^*−*^ to ensure electrical neutrality^57,58^. Models for the A450P and L462P mutants were manually derived from this assembly. The initial simulation cells measured 116 *×* 114 *×* 133 Å^3^ in volume.

The simulations were driven by NAMD2.14 code^59^, which uses a fully periodic BBK-type integrator with velocity rescaling^60,61^. We employed rigid bond constraints^62,63^ to achieve numerical stability at a timestep of *δt* = 2 fs^64^. Our NVT calculations applied a Langevin thermostat to heavy atoms for temperature control (*T* = 300.0 K; damping *γ* = 1.0 ps^*−*1^)^61^, while NPT simulations controlled pressure with a Langevin piston (target *P* = 101.325 kPa; period = 100.0 fs; decay time = 50.0 fs)^65^. Cell fluctuations transverse to the bilayer were allowed while maintaining constant cross-sectional area. Multiple time-stepping was used for nonbonded interactions^66^, with short-range interactions evaluated every 2 fs and full electrostatics every 4 fs. Short-range interactions were cut off at 1.2 nm and smoothed with sigmoidal rescaling for atoms separated by more than 1.0 nm. Smoothed particle mesh Ewald (PME) was used for long-range electrostatics (120 *×* 120 *×* 144 grid)^67–69^. Equilibration was performed in phases. First, the bilayer was ‘melted’ by performing 2000 steps of conjugate gradient (CG) minimization followed by 1.0 ns of NVT equilibration with all non-lipid tail atoms fixed. Next, a harmonic constraint potential (*k*_prot_ = 5 kcal mol^*−*1^ Å^*−*2^) was applied to hold protein atoms near their initial positions, and 2000 steps of CG minimization and 2.0 ns of NPT equilibration was used to relax lipid and solvent. During this process, we applied a transverse force *F* = ±*k*H_2_O *· P* $\hat{z}$ (with *k*H_2_O = 0.1 kcal mol^−1^ Å^−2^) to displace waters from within the lipid bilayer, checking for water residence every 0.5 ps. Finally, all constraints were removed, and production calculations were conducted for 185 ns in the NPT ensemble. Structural samples were captured every 5 ps, and the first 25 ns of the trajectories were discounted as an initial equilibration period to give 160 ns of production simulation.

Dimeric VLCAD was generated by superposing the AlphaFold model on the chains of the crystal structure (PDB ID 3B96) and minimizing the RMSD between matching atoms. The dimer was then oriented along its principal components, providing electrostatic and hydrophobic complementarity with the membrane, and yielding an initial condition placement that was both physically sensible and mechanically stable.

### Statistical methods

Prism 9 software (GraphPad) was used for data analysis and calculating mean and s.e.m. values.

### Biological materials

Plasmids are available upon request to the corresponding author.

### Reporting summary

Further information on research design is available in the Nature Research Reporting Summary linked to this article.

## Supplementary information

Supplementary Information

Description of Additional Supplementary Files

Supplementary Data 1

Reporting Summary

## Data Availability

The data that support this study are available from the corresponding author upon reasonable request. All data generated or analyzed for this study are included in this manuscript, its supplementary information, and source data file. The raw HDX MS data for VLCAD have been deposited to the ProteomeXchange Consortium via the PRIDE partner repository^70^ with the dataset identifier PXD029565. The structure of VLCAD ΔEx3 has been deposited with the PDB under accession code 7S7G. PDB entries 3B96 and 2UXW were also used in the course of this study. Source data are provided with this paper.

## Source data

Source Data

## Materials availability

Plasmids are available upon request to the corresponding author.
