# Supplementary material for: Structural basis for defective membrane targeting of mutant enzyme in human VLCAD deficiency
Source: Nat Commun. 2022 Jun 27;13:3669. doi: 10.1038/s41467-022-31466-2 (PMC9237092; doi:10.1038/s41467-022-31466-2)
Supplement: Supplementary file 1 — Supplementary Information [file 41467_2022_31466_MOESM1_ESM.pdf]

Structural Basis for Defective Membrane Targeting of Mutant Enzyme in Human VLCAD Deficiency

Michelle S. Prew, Christina M. Camara, Thomas Botzanowski, Jamie A. Moroco, Noah B. Bloch, Hannah R. Levy, Hyuk-Soo Seo, Sirano Dhe-Paganon, Gregory H. Bird, Henry D. Herce, Micah A. Gygi, Silvia Escudero, Thomas E. Wales, John R. Engen, and Loren D. Walensky

SUPPLEMENTARY INFORMATION

Supplementary Table 1

Supplementary Figures and Figure Legends 1-10

Supplementary Table 1 Data collection and refinement statistics for the VLCAD crystal structure (PDB ID 7S7G)

RCSB Accession Code	7S7G
**Data Collection**	
Space group	$C222_1$
Cell dimensions	
a, b, c (Å)	75.26, 108.27, 149.65
α, β, γ (°)	90, 90, 90
Resolution (Å)	57.12-1.34 (1.388-1.34)[a]
R_{pim}	0.02097 (0.592)
$I/\sigma I$	15.54 (1.35)
Completeness (%)	99.42 (99.92)
Redundancy	6.6 (6.5)
**Structure Solution**	
PDB entry used for molecular replacement	3B96
**Refinement**	
Resolution (Å)	57.12-1.34
No. of reflections, unique	135,786
R_{work}/R_{free}	0.1464/0.1684
No. of atoms	4,985
Protein	4,326
Ligand/ion	53
Water	606
B factors	
Protein	24.07
Ligand/ion	19.49
Water	38.19
RMSDs	
Bond lengths (Å)	0.015
Bond angles (°)	1.41
Ramachandran plot (%)	
Preferred	98.41
Allowed	1.59
Not allowed	0.00

[a] Values in parentheses are for highest-resolution shell

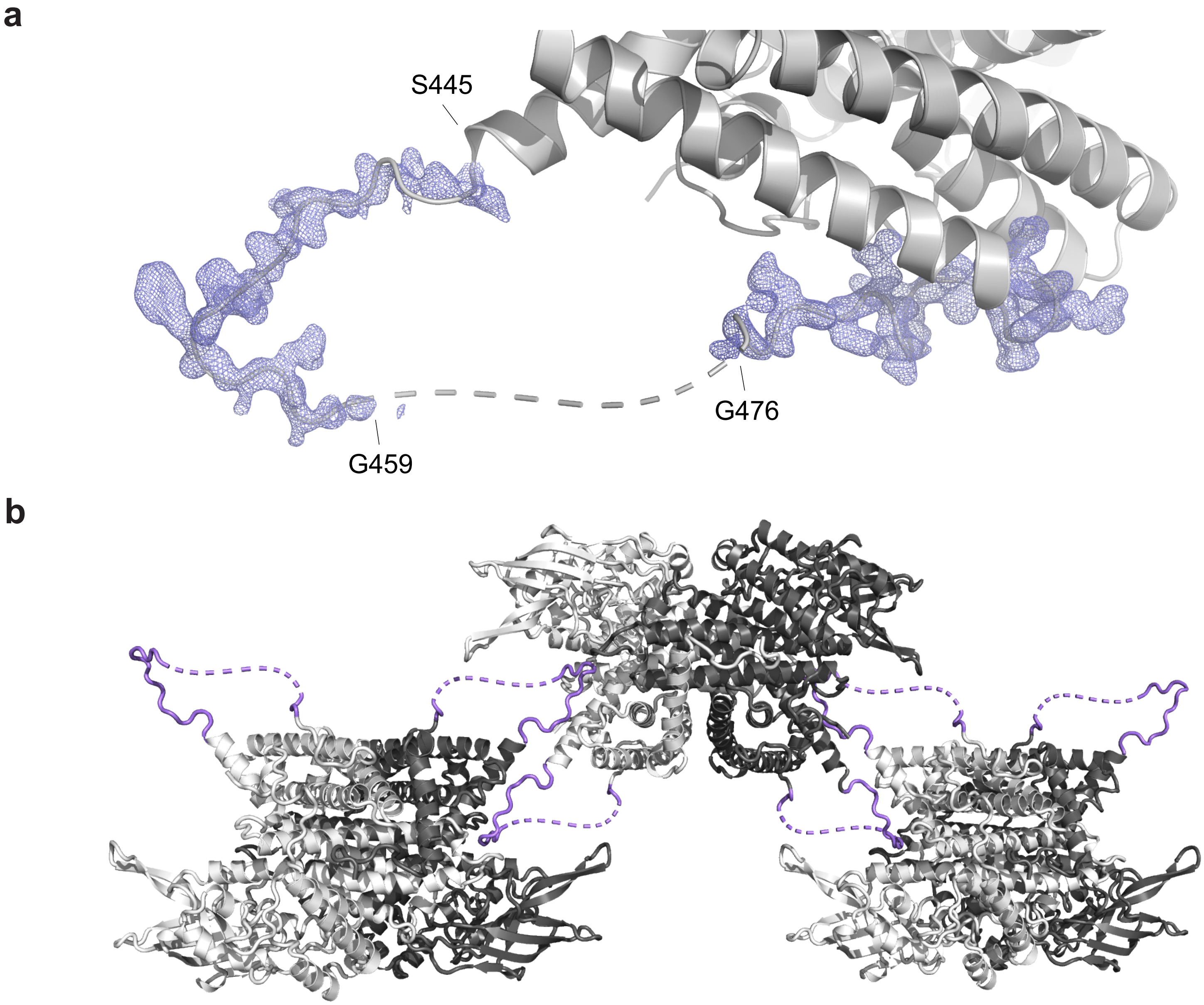

Supplementary Fig. 1

Supplementary Fig. 1 X-ray crystallography of VLCAD. a Electron density map of amino acids within the MBR of VLCAD ΔEx3 (PDB ID 7S7G). **b** Crystal contacts between neighboring VLCAD dimers demonstrate a reciprocal interaction between the MBR of one dimer and the surface groove of another.

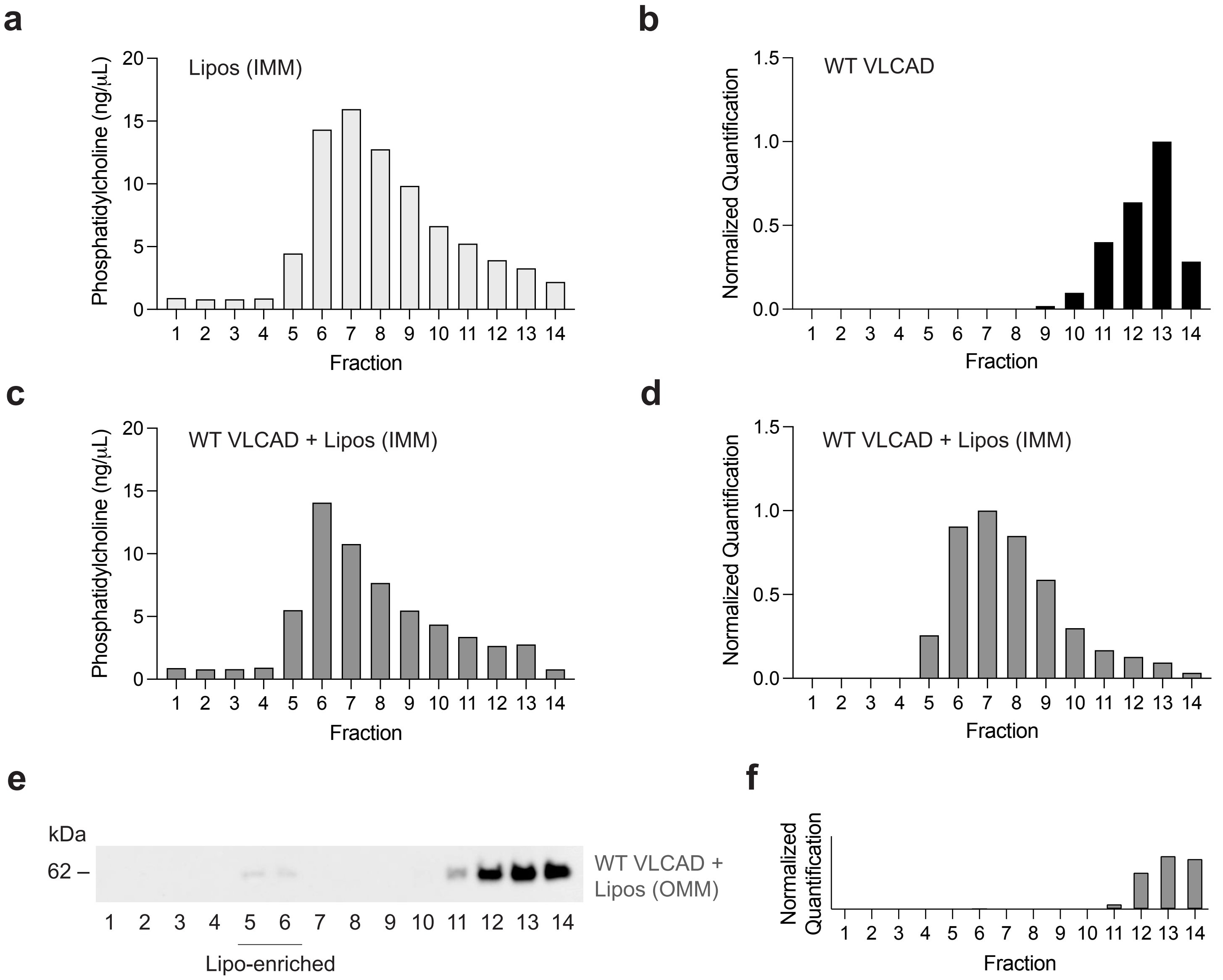
a
Lipos (IMM)
Phosphatidylcholine (ng/μL)
Fraction
b
WT VLCAD
Normalized Quantification
Fraction
c
WT VLCAD + Lipos (IMM)
Phosphatidylcholine (ng/μL)
Fraction
d
WT VLCAD + Lipos (IMM)
Normalized Quantification
Fraction
e
kDa
62 –
WT VLCAD + Lipos (OMM)
Lipo-enriched
f
Normalized Quantification
Fraction

Supplementary Fig. 2 Verification of liposomal translocation fractions and selectivity of VLCAD-membrane interactions. a Quantitation of phosphatidylcholine in SEC fractions from a translocation assay employing liposomes (alone) that reflect the membrane composition of the inner mitochondrial membrane (IMM), as assessed by a Phosphatidylcholine Assay Kit. **b** Quantitation of VLCAD in SEC fractions from a translocation assay employing protein alone, as assessed by densitometry using ImageJ software. **c**, **d** Quantitation of phosphatidylcholine (**c**) and VLCAD (**d**) in SEC fractions from a translocation assay employing a mixture of liposomes (IMM) and VLCAD protein. **e** Liposomal translocation of wild-type VLCAD protein using liposomes that mimic the lipid composition of the outer mitochondrial membrane (OMM), as monitored by VLCAD western analysis of SEC fractions. The experiment was repeated twice with independent preparations of VLCAD protein with similar results. **f** Quantitation of VLCAD observed in liposomal translocation assay fractions (**e**) by densitometry using ImageJ software.

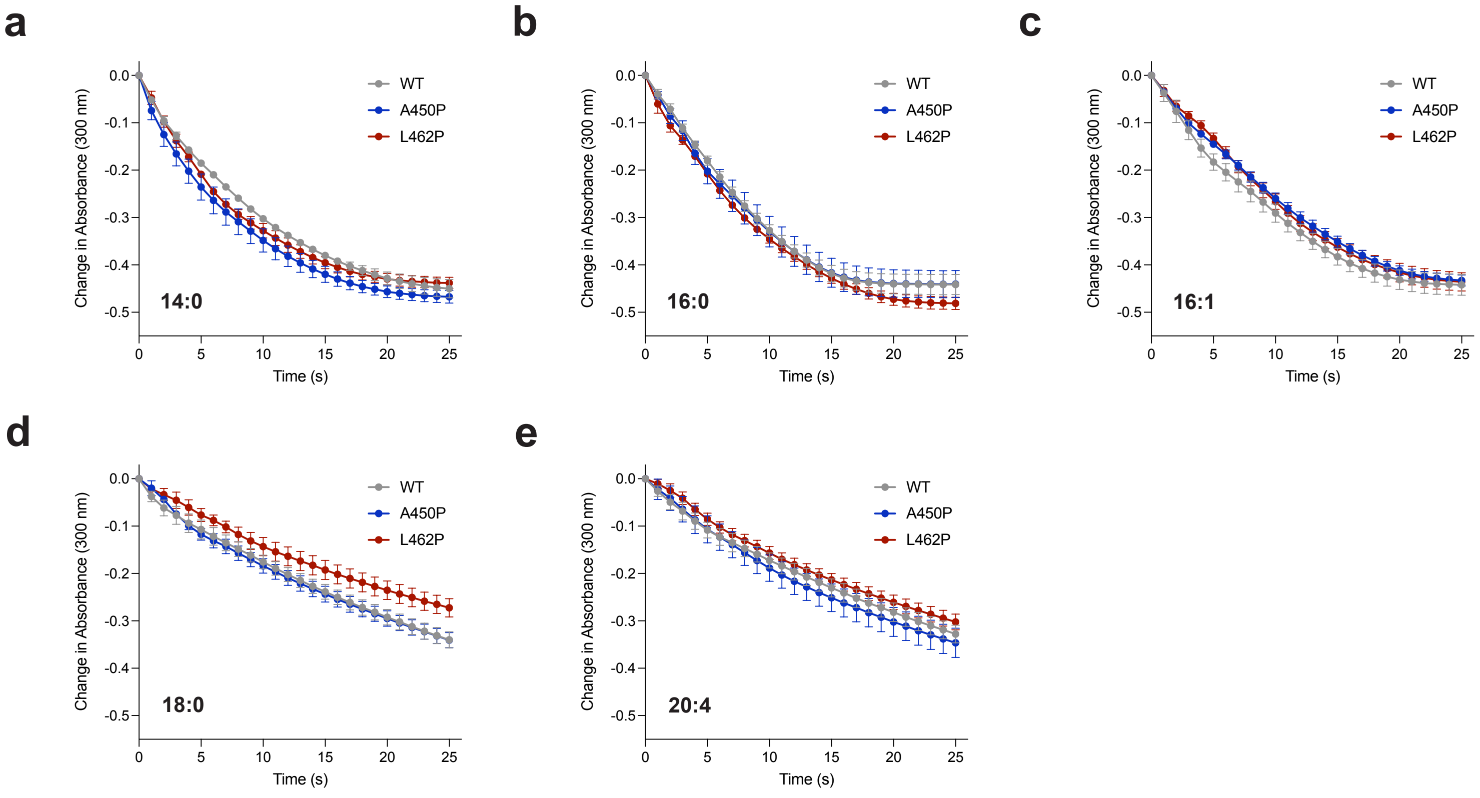

Supplementary Fig. 3

Supplementary Fig. 3 Comparative enzymatic activity of wild-type, A450P, and L462P VLCAD proteins. a-**e** Decrease in absorbance at 300 nm over time for oxidative reactions catalyzed by wild-type, A450P, and L462P VLCAD proteins using 14:0 (**a**), 16:0 (**b**), 16:1 (**c**), 18:0 (**d**), and 20:4 (**e**) long-chain fatty acyl-CoA substrates. Data are mean ± s.e.m. for experiments performed in technical quadruplicate and repeated twice with independent preparations of assay reagents with similar results.

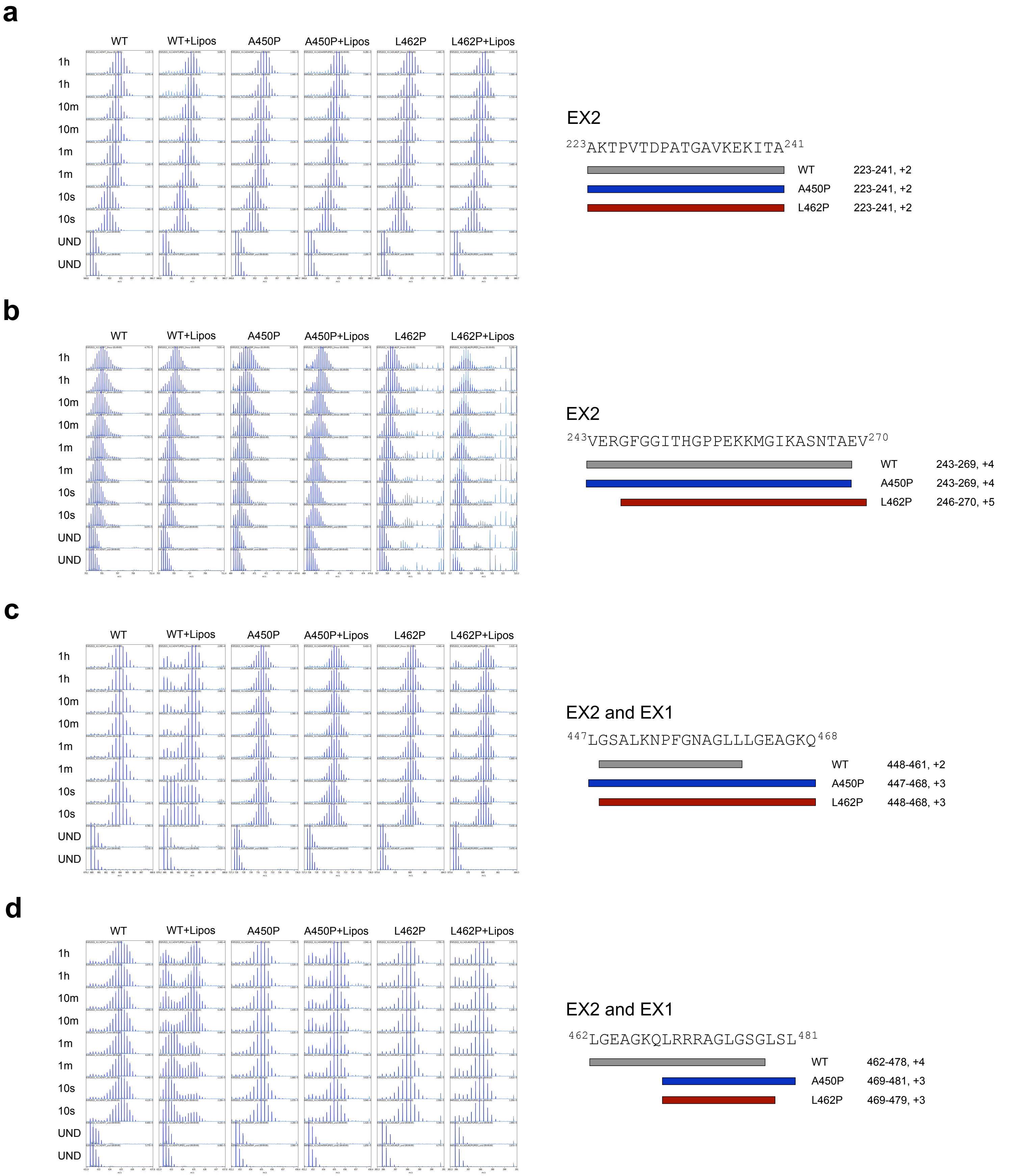

Supplementary Fig. 4

Supplementary Fig. 4 EX2 and EX1 kinetic signatures of representative peptides from the HDX MS analyses. a, **b** Example mass spectra of EX2 kinetic signatures, as observed across almost all VLCAD peptides, for the indicated VLCAD protein (wild-type, A450P, L462P) in the presence or absence of liposomes. The peptide maps (right) demonstrate the residue numbers and charge state of each ion. **c**, **d** Mass spectra of EX2 and EX1 kinetic signatures for peptides covering the 450-479 amino acid region of the MBR of the indicated VLCAD protein (wild-type, A450P, L462P) in the presence or absence of liposomes. EX1 kinetics are prominent for wild-type VLCAD in the presence of liposomes. Relatively minor EX1 kinetics are observed for the A450P and L462P variants in the presence of liposomes. All other conditions demonstrate exclusively EX2 kinetic signatures. The peptide maps (right) demonstrate the residue numbers and charge state of each ion.

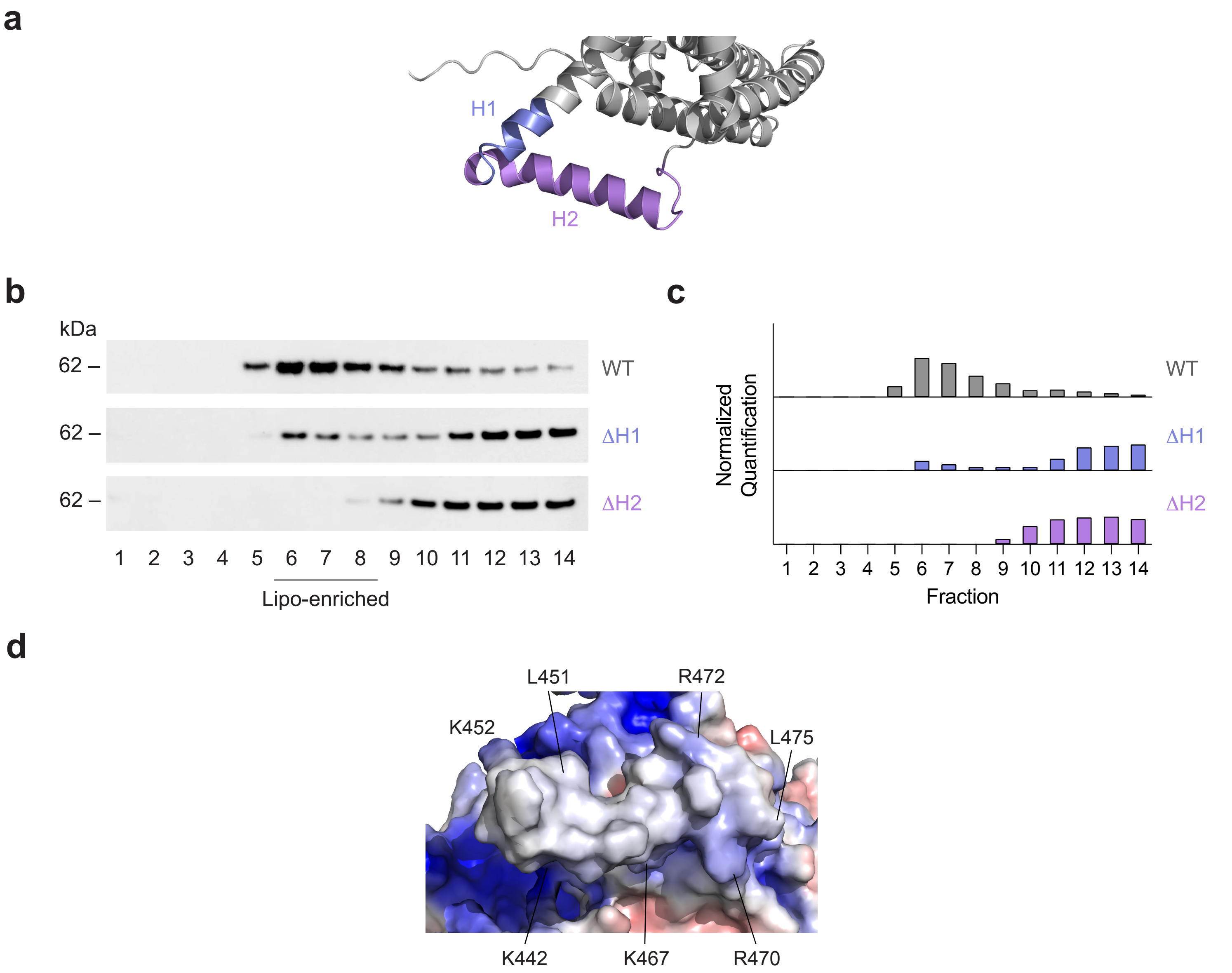

Supplementary Fig. 5

Supplementary Fig. 5 Alternate deletion of MBR substructures impairs VLCAD membrane interaction. a AlphaFold model structure of VLCAD demonstrating helix 1 (H1) and helix 2 (H2) regions of the putative α-helical hairpin subjected to individual deletion for membrane translocation analysis. **b** Comparative liposomal translocation of wild-type, ΔH1, and ΔH2 VLCAD proteins, as monitored by VLCAD western analysis of SEC fractions. The experiment was repeated twice with independent preparations of VLCAD proteins with similar results. **c** Quantitation of VLCAD observed in liposomal translocation assay fractions (**b**) by densitometry using ImageJ software. **d** Electrostatic potential map of the predicted α-helical hairpin (blue, positive; red, negative).

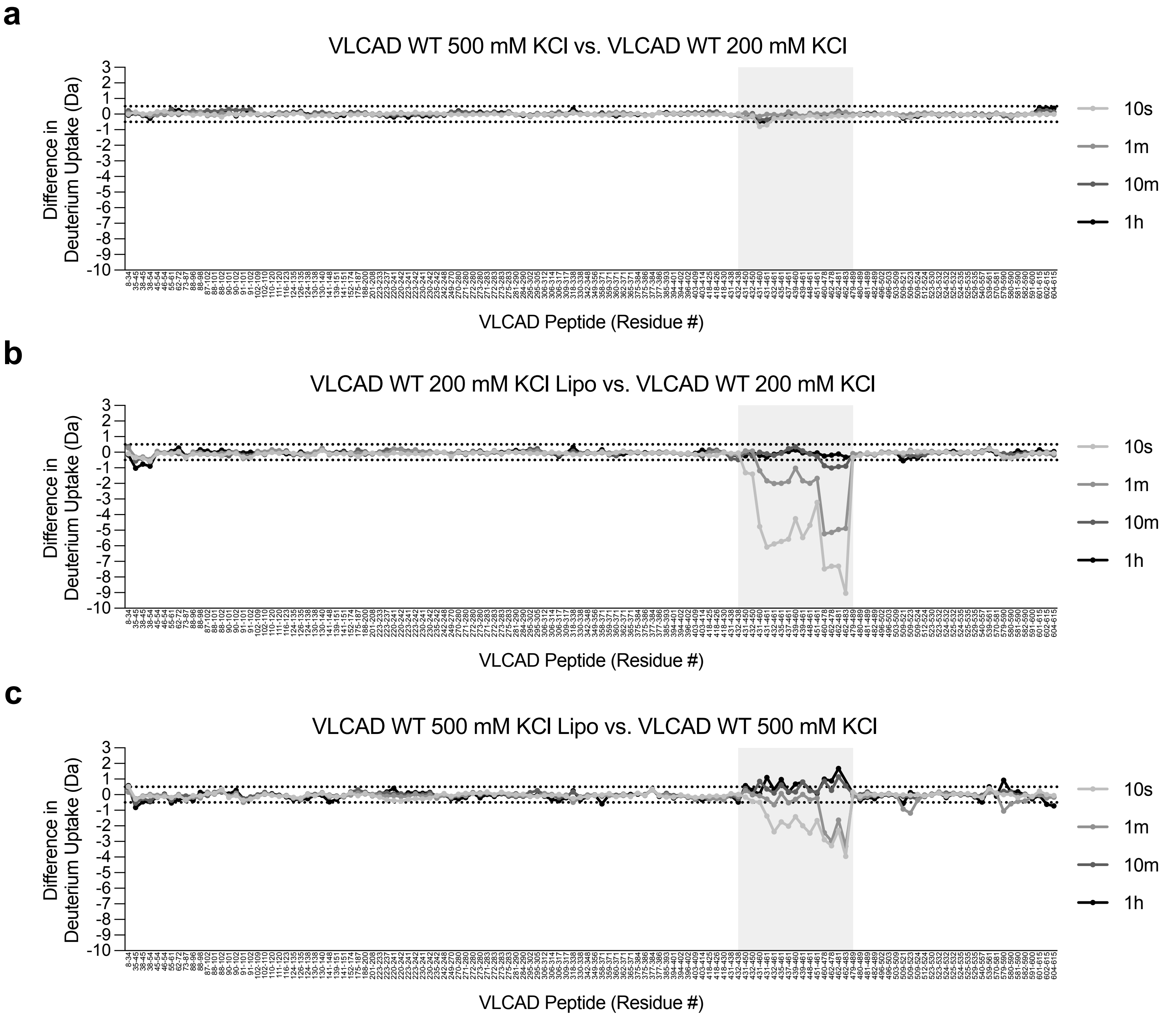

Supplementary Fig. 6

Supplementary Fig. 6 Effect of osmolyte concentration on the HDX MS profile of VLCAD in the presence and absence of liposomal membranes. a-c Deuterium difference plots showing the relative deuterium incorporation of wild-type VLCAD in solution at 500 versus 200 mM KCl (**a**), in liposomes versus solution at 200 mM KCl (**b**), and in liposomes versus solution at 500 mM KCl (**c**). For each comparison, deuteration was measured after 10s, 1m, 10m, and 1h of labeling. Regions of deprotection and protection above 0.5 Da (dotted line) are considered meaningful. The peptides of the VLCAD C-terminal region are highlighted by grey shading. HDX MS experiments were performed twice with independent preparations of VLCAD protein. See Supplementary Data 1 for the HDX MS data used to create this figure.

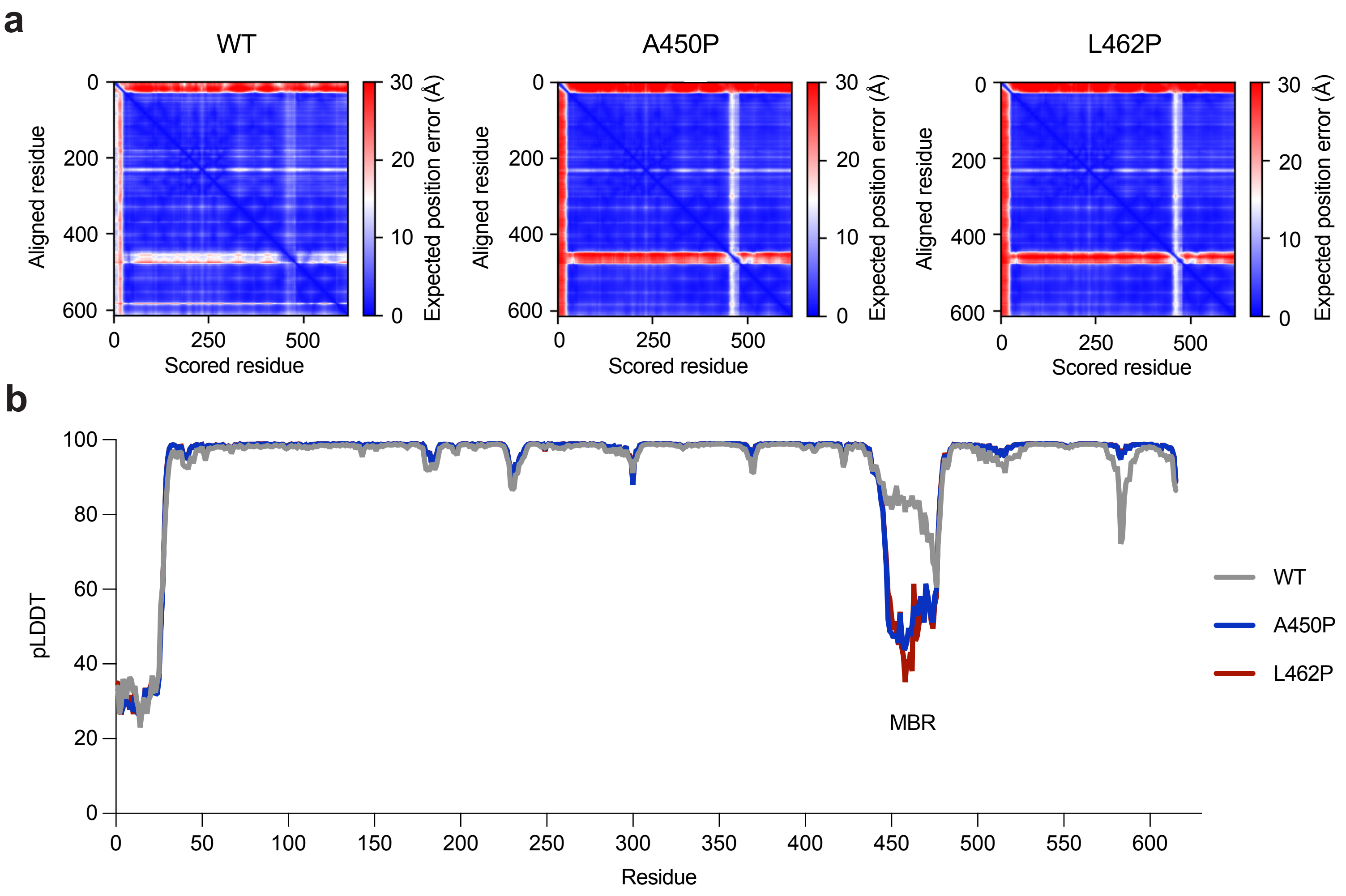
a
WT
A450P
L462P
Aligned residue
Scored residue
Expected position error (Å)
0
200
400
600
250
500
10
20
30
b
pLDDT
Residue
MBR
WT
A450P
L462P
0
20
40
60
80
100
50
100
150
200
250
300
350
400
450
500
550
600

Supplementary Fig. 7 Quality statistics for AlphaFold models of wild-type, A450P, and L462P VLCAD proteins. a, **b** Predicted aligned error (**a**) and per-residue confidence scores (pLDDTs) (**b**) for wild-type, A450P, and L462P VLCAD AlphaFold models.

a

```
WT:     KGKELSGLGSALKNPFGNAGLLLGEAGKQLRRRA
A450P:  KGKELSGLGSPLKNPFGNAGLLLGEAGKQLRRRA
L462P:  KGKELSGLGSALKNPFGNAGLLPGEAGKQLRRRA
```

b

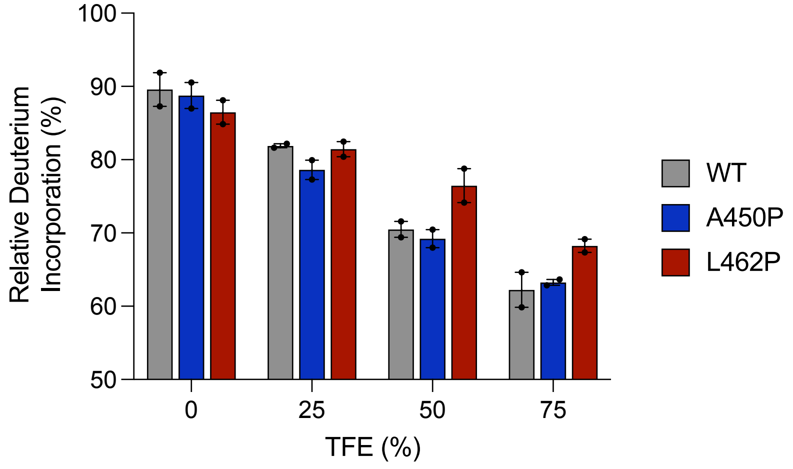

Supplementary Fig. 8

Supplementary Fig. 8 HDX MS analysis of synthetic peptides corresponding to the membrane binding region of VLCAD. a Amino acid sequences of wild-type, A450P, and L462P VLCAD peptides (aa 440-473). **b** The relative percent deuteration of wild-type, A450P, and L462P peptides after 10 seconds of deuteration in different amounts of TFE. Data are mean ± s.e.m. for HDX MS experiments performed twice with independent preparations of VLCAD peptides.

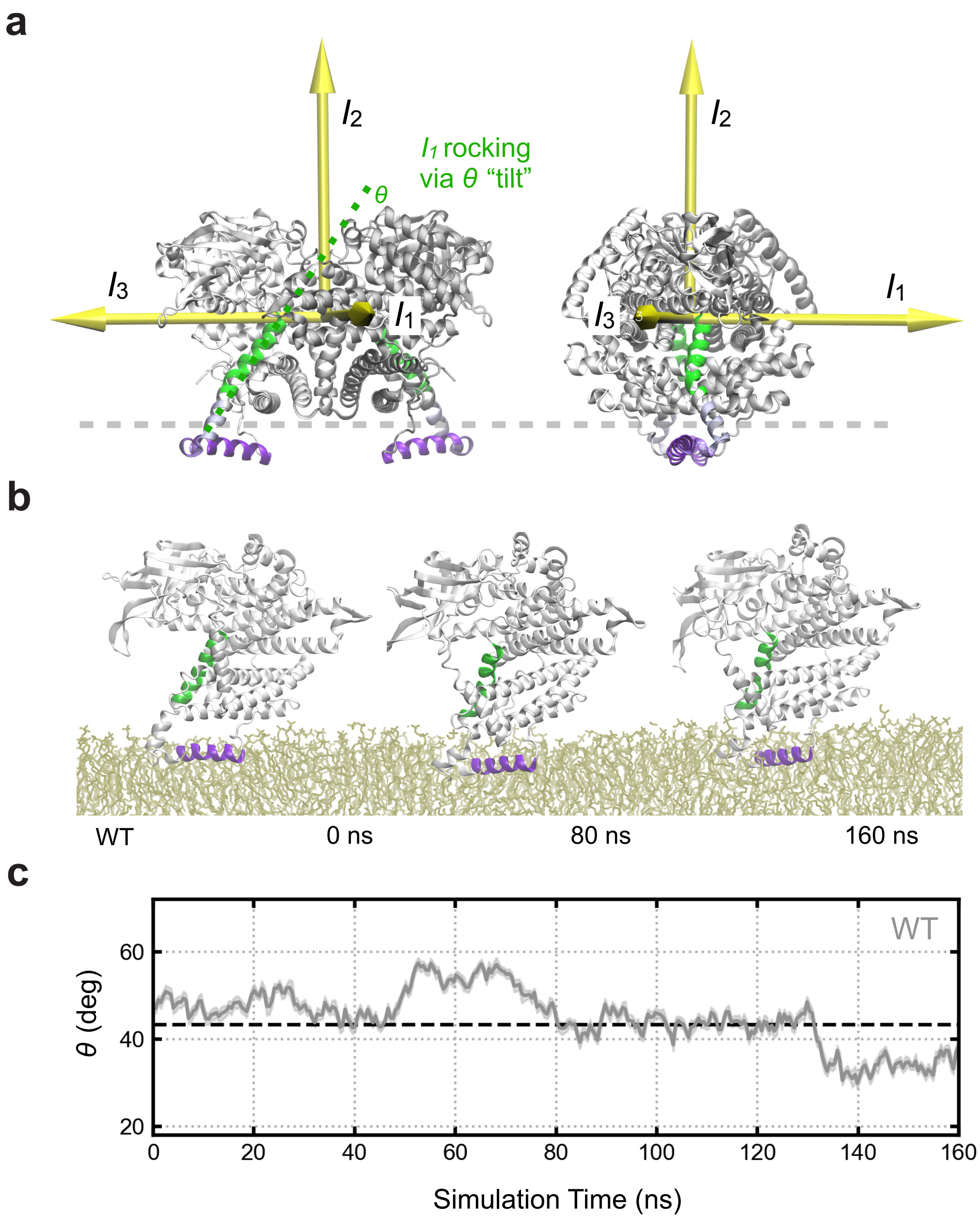
a
l_2
l_1 rocking via θ "tilt"
θ
l_3
l_1
l_2
l_3
l_1
b
WT
0 ns
80 ns
160 ns
c
WT
θ (deg)
60
40
20
0
20
40
60
80
100
120
140
160
Simulation Time (ns)

Supplementary Fig. 9 Initial orientation of membrane-bound dimeric VLCAD mimics that of the simulated monomer. a Initial positioning of dimeric VLCAD in contact with the model membrane. Principal moments of inertia (I_1, I_2, and I_3) are demonstrated by yellow arrows and define a basis for rigid-body motion of the construct. The angle θ corresponds to the rocking motion of an index helix (colored green) about the first principal moment. **b** Representative poses of monomeric VLCAD throughout the simulation, highlighting the relative stability of the protein-membrane configuration. **c** Values of θ over the course of the simulation of monomeric VLCAD reveal minimal rocking and consistently track with the initial positioning of dimeric VLCAD (indicated by the dashed line). The reference θ was obtained by aligning the second principal moment of the dimer with the bilayer normal. The angle θ is defined in terms of the smallest principal moment of the index helix (green) and a plane formed by the lipid head group phosphates. The trajectory for monomeric VLCAD was smoothed by averaging values of θ over 0.5 ns bins, and bands reflect the standard deviation within these bins.

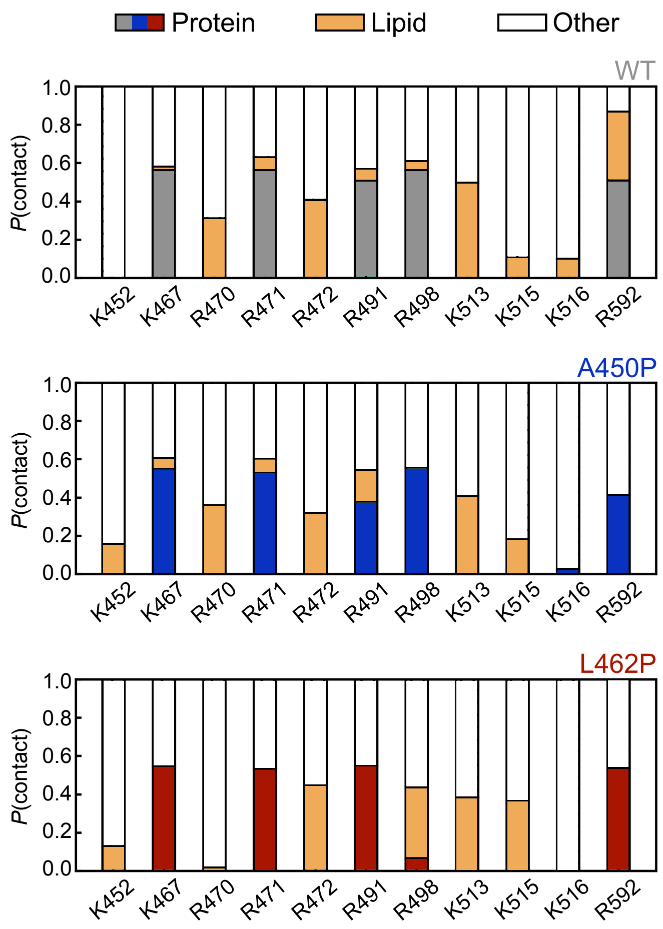
Protein
Lipid
Other
WT
A450P
L462P
P(contact)
1.0
0.8
0.6
0.4
0.2
0.0
K452
K467
R470
R471
R472
R491
R498
K513
K515
K516
R592

Supplementary Fig. 10 Contributions of exemplary cationic residues to VLCAD-membrane interaction. Contact probabilities *P* for cationic residues of the VLCAD C-terminal region with salt-bridging anionic residues within the protein (grey, wild-type; blue, A450P; red, L462P), negatively charged cardiolipin headgroups of the lipid bilayer (orange), or other orientations (white). A contact is defined by an interaction distance of $\leq$ 4.0 Å between the sidechain nitrogen of a cationic residue and the charged oxygen of an anionic amino acid sidechain or cardiolipin phosphate. Statistics are accrued for the last 80 ns of simulation. Alteration of the VLCAD-membrane interaction network upon proline mutagenesis is reflected by the observed shifts in probability of protein and lipid contacts for discrete cationic residues. For example, the loss of lipid binding by both proline mutants at R592, a residue that lies relatively distant from the lipid headgroups, suggests a change in overall orientation, such as a tilt, which disrupts membrane contact.
