# Supplementary material for: Structural basis for defective membrane targeting of mutant enzyme in human VLCAD deficiency
Source: Nat Commun. 2022 Jun 27;13:3669. doi: 10.1038/s41467-022-31466-2 (PMC9237092; doi:10.1038/s41467-022-31466-2)
Supplement: Supplementary file 2 — Description of Additional Supplementary Files [file 41467_2022_31466_MOESM2_ESM.pdf]

Description of Additional Supplementary Files

File Name: Supplementary Data 1
Description: Comprehensive experimental details and parameters, proteolytic maps for all proteins, and the raw numeric values used to generate HDX MS figures.
